# A suggestive approach for assessing item quality, usability and validity of Automatic Item Generation

**DOI:** 10.1007/s10459-023-10225-y

**Published:** 2023-04-25

**Authors:** Filipe Falcão, Daniela Marques Pereira, Nuno Gonçalves, Andre De Champlain, Patrício Costa, José Miguel Pêgo

**Affiliations:** 1 Life and Health Sciences Research Institute (ICVS), School of Medicine, University of Minho, Largo Do Paço, 4710-057 Braga, Portugal; 2 ICVS/3B’s, PT Government Associate Laboratory, Braga, Guimarães, Portugal; 3 iCognitus4All – IT Solutions, Braga, Portugal; 4 AstraZeneca, Gaithersburg, USA

**Keywords:** Automatic Item Generation, Item quality, Item writing, Usability, Validity

## Abstract

Automatic Item Generation (AIG) refers to the process of using cognitive models to generate test items using computer modules. It is a new but rapidly evolving research area where cognitive and psychometric theory are combined into digital framework. However, assessment of the item quality, usability and validity of AIG relative to traditional item development methods lacks clarification. This paper takes a top-down strong theory approach to evaluate AIG in medical education. Two studies were conducted: Study I—participants with different levels of clinical knowledge and item writing experience developed medical test items both manually and through AIG. Both item types were compared in terms of *quality* and *usability (efficiency and learnability)*; Study II—Automatically generated items were included in a summative exam in the content area of surgery. A psychometric analysis based on Item Response Theory inspected the validity and quality of the AIG-items. Items generated by AIG presented quality, evidences of validity and were adequate for testing student’s knowledge. The time spent developing the contents for item generation (cognitive models) and the number of items generated did not vary considering the participants' item writing experience or clinical knowledge. AIG produces numerous high-quality items in a fast, economical and easy to learn process, even for inexperienced and without clinical training item writers. Medical schools may benefit from a substantial improvement in cost-efficiency in developing test items by using AIG. Item writing flaws can be significantly reduced thanks to the application of AIG's models, thus generating test items capable of accurately gauging students' knowledge.

## Introduction

The majority of knowledge tests used in medical education, within various medical content areas, are based on multiple-choice questions (MCQ). While MCQs are effective in gauging student’s knowledge, developing them demands in depth-comprehension of both the subject-matter and the purpose of the assessment, which makes them time-consuming and expensive to write. Automated Item Generation (AIG) is a model-based next-generation assessment method where computer algorithms generate items together with estimates of their psychometric parameters. It is a contemporary method that can scale the item development process for medical schools, producing large numbers of high-quality items both quickly and efficiency. Despite this promising framework, the item quality, usability, and validity of items generated by AIG relative to those manually written remains generally unexplored. Through a multimodal strategy based on the evaluation of medical test items developed by participants with varied levels of clinical expertise and item writing experience (ranging from no training to professional practice), as well as on the psychometric analysis of the item parameters of a medical summative exam, we aim to: (i) compare the quality of AIG-items vs manually written items (*item quality*); (ii) measure the efficiency of AIG and explore its learnability properties (*usability*) and; (iii) search for sources of evidence that support AIG as a valid item development method (*validity*). Our work contributes to the literature by assessing whether AIG can meet the quality, usability and validity standards expected of traditional testing methods that are used in educational assessment.

### Background

#### The role of multiple-choice questions in medical education

The most popular tool for testing in medical education are exams comprised of multiple-choice questions (MCQs) (Douthit et al., 2021; Grainger et al., 2018). Besides allowing for instant scoring, MCQs are suitable for achievement testing since they cover different skills and enable the assessment of numerous candidates in a cost and resource efficient manner (Royal et al., 2018; Rust et al., 2020).Writing MCQs is complex since experts must outline and replicate cognitive problem-solving skills (Billings et al., 2020; Gierl et al., 2012). In order to plan this procedure, experts must share their knowledge and collaborate behind the scenes. Manual (or traditional) item writing involves at least five stages: (i) hiring item writers to broaden the approaches of content experts (Sinharay et al., 2003); (ii) training item writers to develop new items; (iii) detailed reviews; (iv) empirical try-outs to calibrate item parameters (de Chiusole et al., 2018; Embretson & Kingston, 2018) and; (v) the inclusion of new items in operational exams based on blueprint specifications and empirical properties (Embretson & Kingston, 2018).

This complex set of procedures adds expense and time to the item development process, limiting the availability of items needed for proper assessment (Gierl & Haladyna, 2012). Although they are feasible for lower-stakes exams, these approaches are less appealing for large-scale exams (Gierl et al., 2012). The limitations of this procedure are even more pronounced when large number of items are needed to build item banks—pools of items containing information about their content and psychometric details—or parallel test parcels, where each item is treated as an isolated entity (Gierl et al., 2022a; Gierl & Lai, 2013a). Since each item is designed, reviewed and formatted individually, unanticipated psychometric results may arise due to spurious elements that were missed throughout the development process (Mindyarto et al., 2018). Additionally, since exams are continuously administered, item exposure becomes a concern over time (Sinharay et al., 2003). Consequently, it would take thousands of MCQs to develop secure item banks (Gierl & Lai, 2013a). The restocking of new items is essential for assuring quality assessment, especially in high-stakes testing (Embretson & Kingston, 2018). As educators face pressure to develop large numbers of new items, alternative methods of item development become necessary (Gierl & Lai, 2013a; Jozefowicz et al., 2002).

#### Automatic item generation as a solution

In order to address the drawbacks of conventional item writing, modern psychometrics research explored the use of computer algorithms to construct test items (Falcão et al., 2022; Harrison et al., 2017). The use of algorithms is novel and might potentially yield an infinite number of items for assessment (von Davier, 2018). Among the approaches, *Automatic Item Generation* (AIG) refers to the process of employing cognitive models, i.e. structured content reflecting the sources of information that underly the cognitive process, to generate test items with the aid of computer technology (Gierl et al., 2022a). It represents an emerging cutting-edge assessment development method that mixes human experience with computer algorithms, promising to generate vast amounts of new items in a short time from a single model (Choi, 2020; Gierl et al., 2021; Gierl et al., 2022a). Simply stated, AIG may be conceptualized of as an approach where algorithms generate items along with estimates of their psychometric parameters due to the merging of cognitive and psychometric theory (Choi, 2020; Kosh et al., 2019; Mindyarto et al., 2018).

**Fig. 1:**
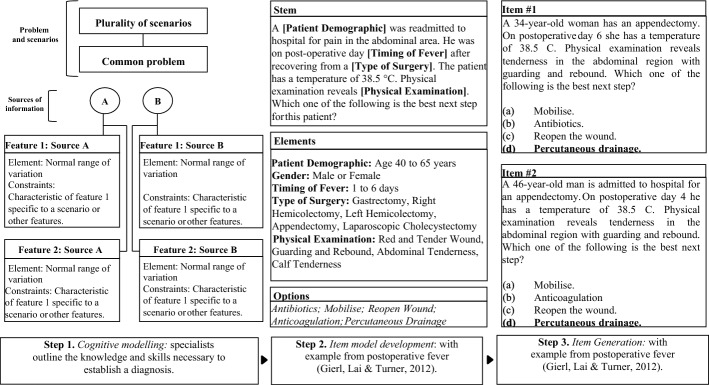
Three-step process for generating medical MCQs based on AIG

The generation of medical MCQs using AIG is predicated on a scalable content development method based on a strong theory-driven approach in which a three-stage process generates items considering clinical scenarios (Cf. Figure 1). In the first stage, the content for item generation is identified through a cognitive model (Gierl & Lai, 2016). Developing this structure requires the selection of a topic from the test blueprint and a content expert to summarize a typical approach to make a medical diagnosis (Gierl & Lai, 2016). During this process, the cognitive and content specific informations needed to solve the problem are organized into a coherent structure that contains the content relationships and information sources necessary to formulate a medical diagnosis in a specific content area (Gierl et al., 2022a; Lai et al., 2016). These models list cognitive and content-specific information, characterize higher-order versus lower-order forms of thinking to solve problems, and provide a theoretical understanding of test performance (Gierl & Lai, 2016; Leighton & Gierl, 2011). Once the cognitive models are determined, the stimulus features will be determined in accordance with these models (Yang et al., 2022). In the second stage, an item model is designed to outline where the content from the cognitive model should be placed in order to generate new items (Gierl et al., 2022a). This model will specify which parts of the item can be manipulated, including the context, content, and the question that examinees are required to answer (Gierl et al., 2012). For a typical MCQ, it includes the stem (part of the item model that compiles the information needed to solve the problem), response options (all answers, including distractors and correct options), the lead-in-question and other ancillary information (Gierl et al., 2022a). Finally, in the third stage, algorithms integrate the content from the cognitive model outlined in step 1 into the item model developed in step 2 (Gierl et al., 2022a). Computer algorithms are used to conduct this process because the assembly often involves challenging combinatorial operations (Gierl et al., 2022a). The generation procedure can be viewed as an iterator that permutes over all possible value combinations, removing any that do not adhere to the restrictions previously established in the cognitive model (Gierl et al., 2022a).

Unlike conventional item development methods, where each item is treated as the unit of analysis and is created individually, AIG treats the model as the primary unit of analysis where a single model is utilized to generate numerous items (Gierl et al., ). Consequently, item development is linked to the number of available models rather than the number of experts (Gierl et al., 2022a). Through this approach, it is possible to specify and manage item features (i.e., the elements in the assessment task that must be manipulated) that predict test performance since cognitive features are defined in great detail. As a result, the generated items can potentially be calibrated without the need for extensive field or pilot testing because it is possible to specify the criteria that influences item difficulty (Gierl & Lai, 2012; Leighton & Gierl, 2011). Importantly, the cognitive models integrate aspects of active learning methologies and theoretical constructs that are thought to underpin the thought process of clinical reasoning, such as illness scripts (Schmidt & Mamede, 2015). Additionally, AIG may significantly help faculty members avoiding common item constrution errors. Due to the fact that the majority of faculty members have little to no formal training in item construction, adopting standardized templates such as the ones used within cognitive model framework that AIG uses for item development can also help them improve their item writing skills and generate more precise assessments tools of student’s knowledge (Royal et al., 2018).

#### AIG versus manual item writing

AIG differs from more traditional item development methodologies in several aspects. First, AIG is more intensive in regard to its development and revision processes. Since authors must set values for substitutions in item models and provide instances of item stems and distractors to ensure proper generation of new items, it stands to reason that AIG may require more time/skills than manual item writing for initial item development. Second, the technological environment must also be considered. Since AIG employs algorithms to generate items, this may require more effort from software engineers and subsequent quality evaluations than manually written items. However, the main difference between both processes lies in the fact that traditional item writing is usually based on “single instances”, often without reference to a broader model. AIG, in fact, requires a more naturalistic approach to a clinical problem that is carefully defined based on a theoretical strong framework (Kosh et al., 2019; Pugh et al., 2016), evolving from the symptom/presentation to the clinical manifestations that characterize the conditions that are compatible with it.

Even with these limitations, AIG yields significantly more benefits than manual item writing. Its main asset lies in the generation of large sets of brand-new items, avoiding time-consuming processes typical of manual item writing and promising great resources for item banks (Harrison et al., 2017; Jendryczko et al., 2020). Nevertheless, research into the item quality, usability, and validity of AIG in comparison to manual item development approaches is still in its infancy. To date, only a small number of operational testing scenarios have used items generated using cognitive modeling procedures (Gierl et al., 2016). It will be challenging to defend the implementation of AIG in medical assessment until this subject is thoroughly investigated (Gierl & Lai, 2013a). Therefore, it is crucial to evaluate both item development approaches in light of the following characteristics:

*Item quality.* There is considerable worry that automatically generated items may not be as high-quality as those created using conventional techniques (Pugh et al., 2020). Furthermore, the quality and the psychometric properties of generated items to multiple-choice testing are mostly undocumented (Gierl et al., 2016; Shappell et al., 2021). Generated items need to be of a high caliber for AIG to be truly useful (Gierl et al., 2022b). The few studies that have compared manually and AIG written items generally support the use of the latter. However, the results provided are not consistent and need further evidence. Gierl and Lai (2013a) submitted AIG-items and manually written items to a panel of medical experts, who blindly reviewed the items for quality. The findings suggested that both item types were comparable on almost all quality indicators. However, the authors discovered that AIG-items could be distinguished from manually written items in terms of quality of the distractors, which were rated more poorly for the AIG-items (Gierl & Lai, 2013a). On the other hand, Pugh and colleagues (2020) found that AIG-items, including distractors, were not perceived as different from items created by manual processes. The primary reason behind this discrepancy in findings can be traced back to the fact that post-2013 AIG-engines were enhanced to generate better distractor relationships (Lai et al., 2016). However, this discrepancy yet reveals a lack of clarity regarding the quality of both item types and suggest a need for research. Furthermore, these studies only compared highly trained item writers that tend to produce high quality items. Such expertise, typical of licensing bodies, does not necessarily generalize at the medical school level.

*Usability (or feasibility of implementation)*. Since AIG is based on a human–computer interaction, it is important to compare it to manual item writing methods in terms of *usability* (Jeng, 2005). Usability pertains to the relationships between users, tasks, and environments and is measured in terms of *efficiency* and *ease of use (learnability)* (Jeng, 2005; Lewis, 2016). Studies comparing both methodologies with respect to usability are scarce and do not generalize to a broader context. Gierl and Lai (2013a) report that their participants were able to generate 9496 items after learning the principles of AIG. Prior to learning, the same participants were able to manually develop 25 items. Similarly, Pugh and colleagues (2016) report that a group of inexperienced item writers was able to develop a complete cognitive model within about 2 h, resulting in a set of 5–10 automatically generated items. In short, the available literature seems to give AIG the advantage in regard to usability (Gierl & Lai, 2013a; Harrison et al., 2017). Although AIG has indeed improved the efficiency of item development (Gierl et al., 2012; Pugh et al., 2020), its learnability properties (i.e., the quantity of items that an individual can generate in a particular period of time considering their item writing experience/knowledge) has not been studied, which is why research is needed.

*Validity*. While technology makes it easier to administer test contents to students, it also raises questions regarding test security and validity (Patel, 2021).Validation processes are key to ensuring the validity of assessment tools and consists of assembling evidence to provide a scientific basis for the interpretation of scores (American Educational Research Association, 2018). Item development is essential to validity assessments because it records the procedures and results required to produce high-quality test content (Gierl et al., 2022b). A fundamental issue for AIG research regards the validation of its processes (Gierl et al., 2022b; Shin, 2021). To date, few research has been devoted to collecting evidence to support the validity of AIG (Falcão et al., 2022; Gierl et al., 2022b; Rafatbakhsh et al., 2020). Since incorporating cognitive models into test design and development is required to support validity arguments for test-based inferences, we have reason to suppose that AIG incorporates validity evidence in its methods (Gierl et al., 2022b; Leighton & Gierl, 2011). Therefore, by obtaining evidences of validity, we would be taking a critical step to support greater use of AIG in educational assessment.

#### A top-down, strong theory approach

AIG promises to ease the item development burden (Arendasy & Sommer, 2007; Gierl & Lai, 2012). However, it is critical to understand its benefits and drawbacks (Bejar et al., 2003). This paper employs a strong theoretical approach to evaluate AIG in medical assessment. Over the course of two complementary studies, we evaluated AIG in terms of quality, usability and validity. Our objectives were as follows: (i) to compare the quality of AIG-items vs manually written items (*item quality*); (ii) to measure the efficiency of AIG and explore its learnability properties (*usability*); (iii) to search for sources of evidence supporting AIG as a valid item development approach (*validity*). Our paper contributes to the literature by demonstrating whether AIG can meet the quality, usability and validity standards expected of conventional item development frameworks that are currently used in medical assessment.

## Methods

### General procedure

In the first study, a group of participants developed items in different areas of surgery through AIG and manual writing methods. Both item development approaches were evaluated in terms of *quality* and *usability*. In the second study, an item sample composed of manually written and AIG-items was included in a summative exam of a general surgery curricular unit of the medical course at the School of Medicine of the University of Minho (EMUM). Both item types were evaluated in terms of *item quality* and *validity*.

### Software

R software (version 4.0.5) and R packages “*Psych*” (Revelle & Revelle, 2015), “*mirt*” (Chalmers, 2012), and “*eRm*”(Patrick et al., 2018) were used to conduct statistical analyses. JASP (version 0.15) and R package “*Ggplot2*” (Wickham et al., 2016) were used for graphical representations of the data.

### Study I

#### Sample

Twenty participants (N = 20) with varied levels of clinical knowledge and item writing experience volunteered to take part in the study. Participants were clinical teachers (n = 4), non-clinical teachers (n = 2), non-teacher clinicians (n = 2), members of the academic staff (n = 5) and students (n = 7) from the EMUM. Six participants (three students, one staff member, one clinical teacher, and one clinician) claimed to be aware of AIG prior to the study.

#### Procedure

The item quality and usability of AIG were compared to manually written items using a multi-methods approach. The investigation was conducted through seminars held by the authors in group sessions of no more than five people. Each participant attended one session. Participants were required to complete four tasks during the course of the seminars:

*Task 1: Manual item development*. Participants manually developed one MCQ in a particular domain of surgery (*jaundice; abdominal pain; shoulder pain; knee pain,* or *hematemesis*). They had access to the internet and other sources of information (e.g., MSD manuals) during this process. The time spent developing the item was recorded. The number of characters in the stem, lead-in-question, and option-set of the item, was tallied.

*Task 2: Cognitive model and item model development*. Participants learned the basics of AIG in a workshop taught by the authors of this paper. After learning the logic and instructions to operationalize the cognitive models, participants were asked to develop one cognitive model in a different surgical domain than the one used in the first task. Time spent developing the cognitive model was recorded.

*Task 3: Item generation.* Participants employed algorithms in the cognitive models to generate new items using comercial proprietary software developed for the purpose of this study (AIG module, *QuizOne*®). Item generation time; the number of items generated; and the number of characters in the stem, lead-in-question and in the option-set of each item were counted.

*Task 4: Survey.* Participants answered a survey related to their *confidence* in AIG and its *usefulness*. The survey assessed different facets of respondents' perceptions regarding their confidence in AIG (software used; use of AIG for their own assessments; developing a cognitive model) and its usefulness (measuring knowledge; understanding clinical reasoning; improving item quality). Participants evaluated the different facets on a scale between one (“*not confident at all”;”not useful at all”*) and five (“*totally confident”; “very useful”*). The survey received responses from all respondents.

**Fig. 2:**
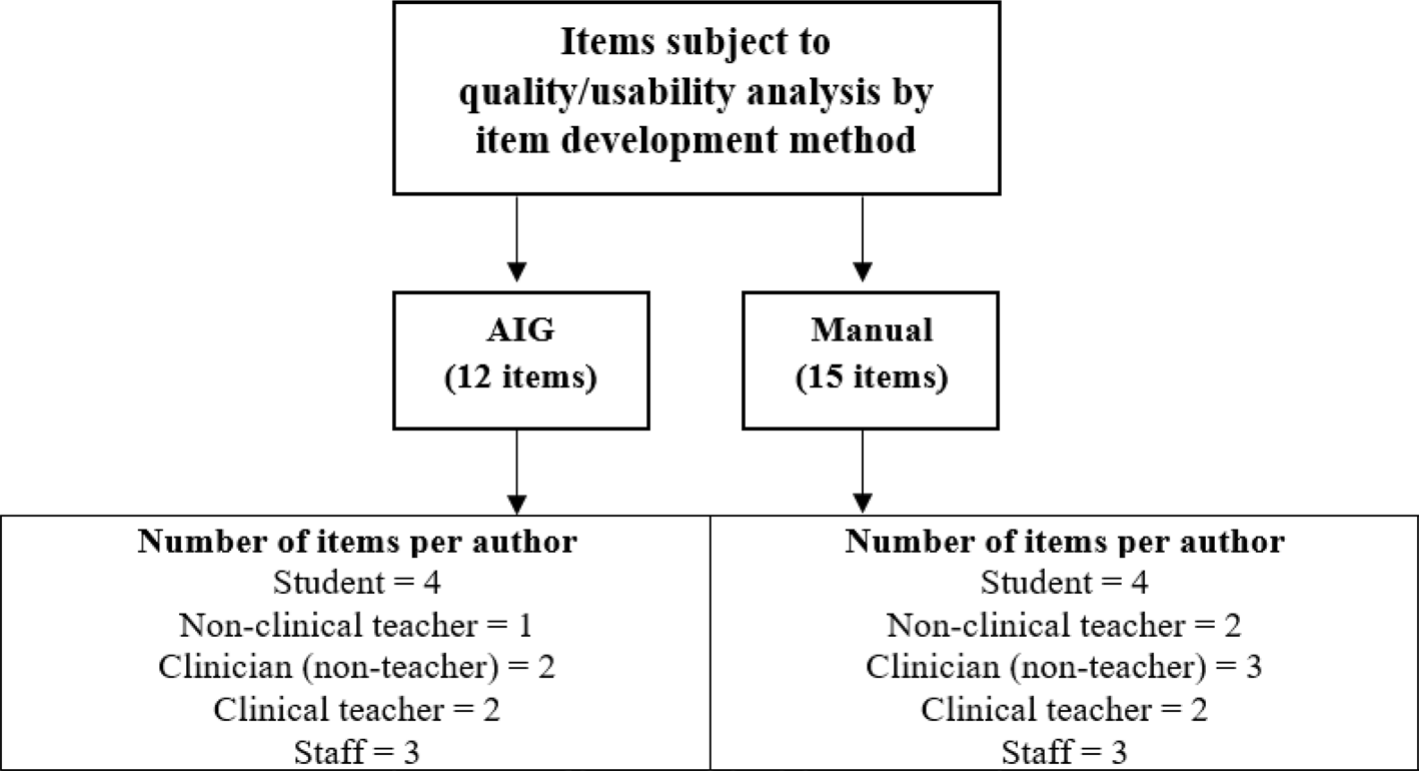
Sample of items used for analysis

After the seminars, a random sample of twenty-seven items (AIG = 12; Manual = 15) developed during the seminars was subjected to a quality and usability analysis. The cognitive models that generated the AIG-items were identified. To ensure that there was no overlap or implausible items during the generation and sampling, all selected items were blind checked for content similarity and plausibility before inclusion in the test form. The method of item development and the author of each item that made up this sample are detailed in Fig. 2. Each of these items was blindly reviewed by a test development expert with experience in reviewing and evaluating MCQs. Judgments from the specialist evaluated the quality of the items (Gierl et al., 2016). The expert did not attend any of the seminars and was not informed about the purpose of the study.

#### Statistical analysis

*Preliminary analysis*. A series of t-tests was performed to determine whether the sample items were comparable in terms of format. This analysis allowed us to verify whether both item types were aesthetically distinct (overall structure) in terms of number of characters in the stem, option-set and lead-in-question, which could bias our results. Independent groups Student t-tests were conducted to test for differences between both item types in regard to the number of characters in the stem. Mann–Whitney tests were computed to test for differences between both item types with respect to the number of characters in the option-set and lead-in-question.

*Item quality.* Items may be linked to evidence that informs judgements of their quality (Gierl et al., 2022b). We used Jozefowicz’s and colleagues (Jozefowicz et al., 2002) item quality rating scheme as a method to measure the *quality* of each question. The rating scheme was designed by its authors to reflect accepted item-writing principles. Each item was rated on a scale from one (“*the item tested recall only and was technically flawed”)* to five (“*the item used a clinical or laboratory vignette, required reasoning to answer, and was free of technical flaws*”). Descriptive statistics were calculated to summarize our data. Mann–Whitney tests were used to see whether there were statistically significant differences in the quality ratings between the two item types.

*Usability (efficiency and learnability)*. Efficiency occurs when outputs are produced with low levels of resources (Johnes et al., 2017). An independent groups Student t-test was used to assess differences in the time spent developing a single item manually vs a cognitive model for AIG. Mann–Whitney tests were employed to assess differences in the generation time and in the number of items developed by both development methods. Learnability was measured using analysis of variance (ANOVAs). As most faculty members in medical schools are required to create their own testing items (Royal et al., 2018; Schmeiser & Welch, 2006), and given that the knowledge and item writing experience of the item writers makes each item unique during its development process (Gierl et al., 2022b), it is relevant to evaluate the learnability of AIG in light of the participants’ item writing experience and clinical knowledge. Considering only the AIG-items, two ANOVAs were conducted: (i) a two-way ANOVA to test for differences regarding the time spent developing a cognitive model, considering the item writing experience (*ItemXP*) and clinical experience/knowledge (*ClinicXP*) of the item author^1^; and (ii) a two-way ANOVA to test for differences in the number of items generated from the models considering the ItemXP and ClinicXP of the author. We retained a nominal type I error rate of 0.05 for all analyses. Data from the surveys were also used to inspect AIG’s learnability. Descriptive statistics were used to summarize the data.

#### Results

*Preliminary Analyses.* No statistically significant differences were found between the number of characters in the stem, *t* (20.7) = 0.196, *p* = 0.846, *d* = 0.072, or in the lead-in-question, U = 109, *p* = 0.338, *r* = −0.184, of both item types. However, statistically significant differences were noted regarding the number of characters in the option-set, *U* = 156, *p* < 0.001, *r* = −0.620, with manually written items presenting more characters (*Mdn* = 119; *IQR* = 283) than AIG-items (*Mdn* = 82.5; *IQR* = 28.75). Considering that both item types were comparable in two out of three components of the selected MCQs, we believe that both item types were comparable and hardly distinguishable.

*Item quality.* The majority of AIG-items yielded high quality ratings (*M* = 4.92; *SD* = 0.289). Only one AIG-item was evaluated with a rating of four. The reason for this score was related to the detection of one item flaw (an implausible distractor). Ratings of manually written items were more variable (*M* = 2.93; *SD* = 1.53). Statistically significant differences in the quality ratings of both item types were found, *U* = 16, *p* < 0.001, *r* = -0.746, with higher quality ratings associated with AIG-items (*Mdn* = 5; *IQR* = 0) vs manually written items (*Mdn* = 4; *IQR* = 3).

*Efficiency*. *S*tatistically significant differences were observed in regard to time spent developing items between both methods, *t*(25) = 6.39, *p* < 0.001, *d* = 2.44. The average time (in minutes) to develop a cognitive model for AIG was higher (*M* = 74.3; *SD* = 19.5) than when manually developing a single new item (M = 31.5; SD = 15.4). Furthermore, when applying a ratio comparison for the two times, we found that the time spent developing a cognitive model was 2.36 times as long as the time devoted to manually writing a single item.

Next, we compared the time algorithms spent generating items from the models vs the time spent by participants manually writing a single item. The Mann–Whitney test revealed statistically significant differences, *U* = 180, *p* < 0.001, *r* = 0.846, with the algorithms producing new items faster (*Mdn* = 0.310; *IQR* = 0.48) than when manually writing a new item (*Mdn* = 27; *IQR* = 27). The time spent manually writing an item was 87.1 times as long as the time spent by the algorithm to generate new items. In conclusion, though it takes more resources to develop a cognitive model than manually writing a single item, the item yield was far more advantageous when using AIG since large sets of items are produced in a relatively short time. Therefore, one should consider AIG as a more efficient item development method.

*Learnability.* A two-way ANOVA was performed to analyse the effect of ItemXP and ClinicXP on the time spent developing a cognitive model. Neither ItemXP nor ClinicXP had a statistically significant effect on the time spent developing a cognitive model (p_ItemXP_ = 0.144; p_clinicalXP_ = 0.216). There was no significant interaction between the effects of ItemXP and ClinicXP (F(4,7) = 0.002 *p* = 0.966, *η*_*p*_^*2*^ = 0). The time spent developing the cognitive models did not vary as a function of the participants’ ItemXP or ClinicXP. This is a point in favour of AIG’s learnability, as inexperienced participants were able to build cognitive models and took approximately the same time as experienced participants to design them.

A second two-way ANOVA analysed the effects of ItemXP and ClinicXP on the number of items generated from the cognitive models. The main effect of ItemXP was significant, F (2, 7) = 12.3, *p* < 0.05, ηp2 = 0.779, suggesting that the number of items generated by novice item writers (M = 393; SD = 439) was greater than experienced (M = 188; SD = 184) or inexperienced item writers (M = 313; SD = 94.4). The main effect of ClinicXP was also significant, F (1, 7) = 13.8, *p* < 0.05, ηp2 = 0.663, suggesting that the number of items generated by non-clinics (M = 469; SD = 443) was greater than clinicians (M = 217; SD = 164). These main effects were qualified by a significant interaction between ItemXP and ClinicXP on the numbers of items generated, F (1, 7) = 16.3, *p* < 0.05, ηp2 = 0.700, suggesting that the effect of ItemXP was greater for non-clinics than clinics. These results again point to the learnability of AIG. ANOVAs results are available in Appendix A.

Participants largely expressed confidence in AIG, according to the survey results. The results support substantial levels of confidence (M = 4.05; SD = 0.759) in the software used to generate new items and moderate levels of confidence (M = 3.89; SD.99) in the use of AIG on their own assessments. However, individuals report having less confidence while creating cognitive models (M = 3.47; SD = 1.02). Participants’ views on the usefulness of AIG were also positive. Participants thought AIG to be a useful tool to measure knowledge (M = 4.42; SD = 0.69); understand clinical processes (M = 4.53; SD = 0.61); and enhance item quality (M = 4.47; SD = 0.70).

### Study II

#### Data collection and sample

Responses to a summative exam in the content area of surgery taken by 132 fifth-year medical students of the EMUM were used as our data. The majority of students were females (73.5%) with ages ranging from 21 to 40, with a mean age of 23 years (*SD* = 3.40).

#### Measure

The exam consisted of 100 dichotomously-scored single best-answer five-option MCQs designed to measure students’ knowledge in surgery. The exam covered multiple surgery content domains (strands): *general and vascular surgery; neurosurgery; urology; otorhinolaryngology* and *ophthalmology*. For the sake of analytic purposes, we only retained the questions from the *general and vascular surgery* strand (28 questions evenly distributed: *Manual* = 15; *AIG* = 13). Final scores were calculated by summing up item scores and dividing by the total number of items.

#### Procedure

Students completed the 2-h exam through a virtual testing platform (*QuizOne*®) under online supervision from their teachers. After finishing the exam, students submitted their responses and the platform closed the respective session.

#### Analytic approach

Attributes of exam items presented as parameter estimates from psychometric models entail evidence regarding the theory on which item construction is based(Bejar, 2012; Zegota et al., 2022). Comparisons between manually written and AIG-items can be used as a validation procedure to ensure that the two item development methods produce comparable outcomes (Gierl et al., 2021).We used the internal structure (i.e., the psychometric characteristics) and performance indices of the items used in the exam as sources of evidence to assess AIG’s validity and item quality (Gierl et al., 2022b; Zegota et al., 2022). Item response theory (IRT) methods were employed to estimate the psychometric properties of the exam items—for a brief overview of IRT, see De Champlain’s work (De Champlain, 2010). We used the Rasch model (RM) (Rasch, 1960) to conduct our analysis due to our sample size, its determinants of item response (respondent’s ability and item difficulty) and its relevance for achievement tests with dichotomous scoring (Hohensinn & Kubinger, 2011).

Prior to formally running the RM, we assessed whether key assumptions were met, namely: (a) *unidimensionality* (i.e., one dimension explaining the covariance among items); (b) *local independence* (i.e., absence of systematic conditional covariance among items); and (c) *monotonicity between latent trait levels (*θ*) and true scores* (i.e., the need of the probability of endorsing an item to increase as the latent ability being measured levels increases). Strategies for evaluating RM assumptions and results are provided in appendix B. After ensuring appropriate model-data fit (Cf. Appendix C) we undertook a calibration process to estimate item properties and help in gauging the quality of the items. Finally, item reliability was assessed using item information function (IIF) plots whereas exam reliability was examined using test information function (TIF) plots and the item reliability index. Statistical procedures were based on the full sample. The significance level was set to α = 0.01 for all analyses.

#### Results

*Item calibration.* Parameter estimates from the fully specified RM are available in Appendix D. Global difficulty estimates of the questions ranged from −2.11 to 2.02 logits (the RM centers on a mean item difficulty value of 0). Item locations did not reveal evenly spaced parameters along the continuum, which may indicate the absence of discrimination between students. This finding is likely due to the fact that we were dealing with medical students, who are known for their high study skills and good academic performances (i.e. restriction of range in candidate ability estimates). The range of difficulty for AIG-items ranged from −2.11 (AIG21) to 1.85 (AIG16) logits, while for manually written items these difficulty parameter estimates ranged from −2.11 (M3) to 2.02 (M10) logits. On average, AIG-items yielded Rasch calibration difficulty levels similar to manually written items, which supports the validity and quality of the AIG process.

**Fig. 3:**
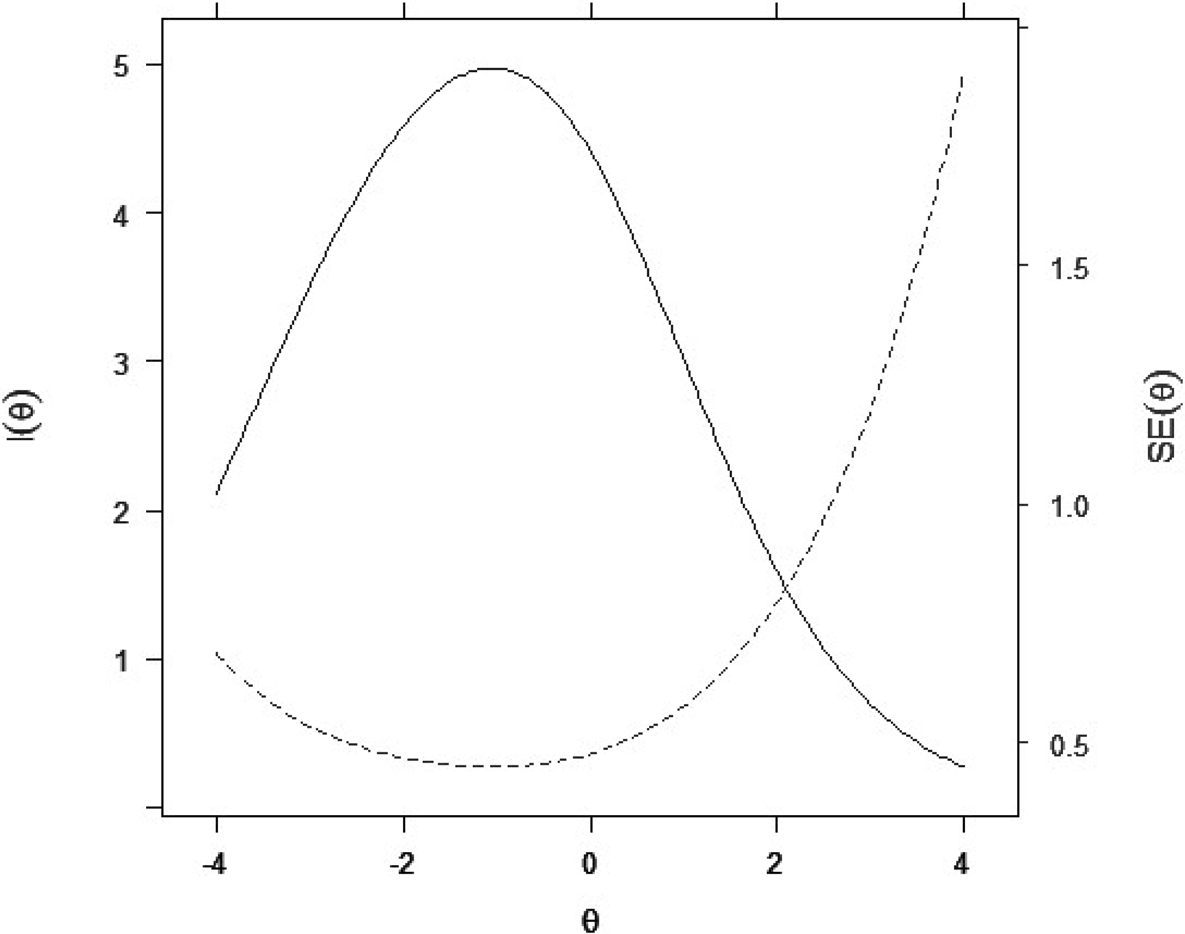
Test information function with conditional standard error of measurement

*Note.* The undashed line represents the TIF. The dashed line represents the conditional standard error of measurement function.

*Item and test reliability.* IIFs revealed that most items conveyed high information values, namely in the middle of the θ distribution (Cf. Appendix E). Since both item types presented similar amounts of information, findings suggest that AIG-items are of high quality and provide precise measurement of candidate ability levels. The TIF was computed together with the conditional standard error of measurement (CSEMs) to assess the level of precision with which the entire exam measured various θ values along the continuum (Cf. Figure 3). The CSEM increased at the high-end of the ability scale, as expected, given the paucity of data located at the extreme ranges of a score distribution. The peak of the TIF (and lowest amount of measurement error or CSEM) was located close to the centre of the ability distribution. Concretely, this suggests that the exam allowed us to measure low-and-middle-ability candidates with the highest level of reliability. The item reliability index was of 0.96, reflecting replicability of the test if applied to another group of comparable subjects.

## Discussion

Despite the fact that most of the work on AIG has been done in theoretical, non-operational contexts, test developers are increasingly employing AIG to augment item banks on practical tests. Naturally, this transition from theory to practice raises unforeseen implementation challenges (Kosh, 2021). Over the course of two studies, we completed a series of comparisons between AIG-items and manually written items to support the implementation of AIG in educational assessment.

### Item quality

Designing quality test items can be challenging given the high standards that they must meet for inclusion into a form, especially in high-stakes testing(Yaneva et al., 2020). Our findings suggest that AIG-items meet these standards, and as such can help to address some of the difficulties associated with item development. We showed that the structure of AIG-items is comparable to that of manually developed items. Manually written items, nonetheless, seem to have a larger number of characters. This may be because cognitive models and algorithms, for the sake of computational power, use a less complex dialogue to allow for the generation of a greater number of items. Literature on the topic (Gierl et al., 2016; Gierl & Lai, 2013a) supports our findings. The results of the item quality assessments conducted so far reveal that, in general, AIG-items are considered to be of similar or superior quality to manually written items.

One concern expressed in the literature around AIG pertains to the quality of distractors (Pugh et al., 2016). Developing distractors is arduous since they must be continually adapted to fit with correct responses based on a rationale within the stem. Users of AIG have thus expressed concerns regarding the quality of distractors generated by AIG (Lai et al., 2016). In our first study, only one AIG-item was flagged due to the detection of an implausible distractor. The remaining distractors were found to be of high quality. Since no differences were found concerning other quality indicators, our results reflect those reported in previous studies, which support the high-quality of items and distractors generated by AIG. Psychometric properties for both AIG and manually written items were also comparable. Difficulty estimates support the use of AIG-items as these properties were similar to those of manually written items. These findings are consistent with earlier research (e.g., ) that have addressed the psychometric characteristics of AIG-items and manually written items. Statistical evidence found revealed that, similarly to manually written items, AIG-items are also capable of producing a wide range of difficulty values. These outcomes highlight the benefit of using a strong theory approach: if cognitive features are identified and the items generated adequate for testing, item features that predict test performance may be specified and controlled (Gierl & Lai, 2012). Consequently, these results encourage the use of of items generated using cognitive modeling processes in operational administrations (Gierl et al., 2016a, 2016b).

### Usability

AIG is not intended to replace human expertise (Gierl et al., 2022b). This is the main reason why we proposed to study the usability of this approach, considering not only its efficiency but also its learnability. Regarding efficiency, not surprisingly, the average time to develop a cognitive model exceeded the average time to write an item manually. However, this difference in time was negated by the rapid generation of hundreds of items from just one cognitive model. As expected, our results confirm past literature in that AIG appears to be an effective alternative for generating hundreds of items based on a single cognitive model in a short period of time (Gierl & Lai, 2013b; Lai et al., 2016). With respect to learnability, time spent developing cognitive models and number of items generated did not vary as a function of the participants' item writing experience or clinical knowledge. Our results are reflective of past literature on this topic, supporting that AIG is an easy to learn technique that does not require much training. Based on the framework used in the design of cognitive models, unexperienced users learned to generate MCQ. This is especially appealing to medical schools and other institutions that may lack the professional training and resources typically at hand with professional licensing bodies.

### Validity

In contrast to the sources of evidence normally employed to evaluate standard item development methods, different types of evidence are needed to evaluate AIG (Gierl et al., 2022b). In the present paper, we used the statistical properties of generated items as a source of evidence of the validity of AIG. The results obtained were promising, as evidence of validity was found. First, ensuring unidimensionality of the item response data was fundamental to assure construct validity. In the same vein, as internal validity concerns item-level psychometric issues such as the relationship of items to a latent dimension and item difficulty, proving that the items were appropriate for both the theoretical concept and the applied psychometric model ensured validity at the item level (Grimm & Widaman, 2012; Shono et al., 2016).

Our work is in line with (Gierl et al., 2022b), as we focused on item statistics to provide evidence that AIG-items can indeed measure the intended knowledge, skills, and competencies of medical students. The evidence found supports AIG as a valid item development method. We believe that this evidence is supported by the strong theory approach that is used for the development of medical MCQs, since the items are designed based on the knowledge, skills and competencies that are the focus of the tests, providing support for the test item content, test item format and possible inferences about the students' domain of interest.

### Implications in the context of education, assessment and test development

School and licensing/certification exams still use a single instance based item writing process that capitalizes on manual, committee based exercises (Albano & Rodriguez, 2018). However, there is a significant shift taking place in the way tests are administered, why they are designed, and who writes them (Gierl et al., 2021). AIG tries to benefit from that expertise in addition to harnessing the power offered by computers for more productive and cost-effective development of test items (Gierl et al., 2021). The use of this methodology has the potential to alter not only the paradigm for how institutions develop test items but also how they manage them. Whereas standard item banks act as an electronic repository for storing, maintaining, and managing information on each item, AIG may employ models rather than items to serve as the unit of analysis in an AIG model bank. Each model, which is written, evaluated, revised, edited, and banked, can be used to produce a large number of questions (Gierl et al., 2022a). Furthermore, the knowledge of content experts is utilised in AIG via the development of cognitive maps whereas the assembly task is done by computers.

The present work contributes two novel sources of evidence to the discussion on AIG. The first point is that while AIG psychometric proprieties have been previously characterized and published in other works, the latter are alsmot uniquevocally based on cognitive maps developed by professional test developers or licensing bodies. In these settings, the quality of items is usually very high even for traditionally developed items. Therefore, AIG may increase efficiency of the process, but the qualitative improvement of items per se is probably limited. The work we completed was in the context of a medical school (MS) that, like many MS, has limited resources. The quality of traditionally developed MCQs is therefore, unsurprisingly, typically lower. We believe that the templates that are used for the development of cognitive models help low-resource institutions to improve not only efficiency but more importantly, they provide a qualitative increment that is much more relevant to this context. A second contribution lies in the fact that we involved participants with very different degrees of expertise in the cognitive model development process. The most “naïve” were neither academicians nor medical students. Rather, some were administrative staff, biomedical researchers, and staff from the communication department. Our belief is that the process underlying the development of cognitive models is transparent and comprehensive, thus allowing participants with little expertise in item writing and limited understanding of clinical sciences to generate high-quality items with ease. In other words, the cognitive model templates impose a format and style of writing that promotes a quick “learning curve” and results in high quality writing. From our review, this issue has very rarely, if ever, been explored in the AIG literature.

Also, not to be neglected, the savings associated with AIG can be significant in comparison to manual item writing. On average, when considering the costs of editing, field-testing and calibrations procedures, the development of a single MCQ for high-stakes testing can range from US$1500 to US$2000 (Kosh et al., 2019; Lai et al., 2009; Rudner, 2010). Given this cost, it is easy to see why item writing is a costly process for test development in the twenty-first century (Lai et al., 2009). Since AIG is an easy technique to learn, institutions will not need to constantly call on professionals to develop test items. Consequently, institutions will save a significant amount of money in development costs, while promoting better assessment.

### Strengths and limitations

The main contribution of our paper lies in the extensive framework that guided the comparisons between AIG and manually written items. These comparisons allowed us to examine whether AIG-items could be applied in the same way as manually written items. The multimodal approach that we adopted is another strong asset, as individual assessment of the items was made based on evaluators' judgments as well as item parameters. Gathering participants' perspective, as we did in our work, is also worth highlighting, as this provides evidence to support a confident application of AIG. In sum, the several sources of evidence collected to support the validity of the AIG process is the main contribution of our work and helps to fill a gap identified in the literature.

However, this work has limitations that should be considered when generalizing our findings to the broader AIG context. First, the samples used in both studies were limited and may lack inferential power. Future research should therefore focus on collecting larger samples of participants with different levels of ClinicXP and ItemXP to support a better generalization of our results. Another limitation of our work rests with the use of only one expert to evaluate the items developed in study I. Using a panel of experts with specific guidelines would provide a more complete and diversified assessment of the items. Similarly, it would be advisable to calibrate our exam items by administering it to a larger group of students. A larger sample size would also allow the application of more robust IRT models capable of estimating additional item parameters that the RM is unable to compute, such as item discrimination and a lower asymptote parameter (“pseudo-guessing”). Finally, we need to underscore that AIG was only evaluated in the content area of surgery. This focus on a single domain is in and of itself a limitation, and applications to other content areas are necessary.

### Directions for future research

AIG still remains a relatively unknown item writing process in practice. For the reasons highlighted in this paper (cost, item quality, etc.), there is a need to explore more innovative and effective means of generating test items (Kurdi et al., 2020). Future studies should focus on assessing the psychometric properties of AIG-items and in particular, on expanding the cognitive model structure to include more complex item types beyond MCQs (Gierl & Lai, 2018). Studies focusing on evaluating the process of learning AIG, the generation of distractors, and how AIG can provide feedback for students while preventing test security breaches would also significantly contribute to the literature. The validation of AIG's processes is another point that can be strengthened with future studies. The item definition, the item development process, and the item quality review also represent useful sources of validity evidence that can be deepened with studies devoted to this topic (Gierl et al., 2022b). The application of AIG in different forms of testing (e.g., computerized adaptive testing) with items with different degrees of complexity (and difficulty) that could be generated on-the-fly would also be worth investigating(Gierl et al., 2021). Finally, it is worth mentioning that cognitive models are still not widely used in large-scale assessments nowadays (Ferrara & DeMauro, 2006; Leighton & Gierl, 2011). As of right now, there are no cognitive models that have a direct relationship to large-scale educational assessments. The dearth of cognitive models might restrain the spread of AIG and support the ongoing use of antiquated methods of development that are becoming more and more out of step with the demands of contemporary evaluation. This entails the need to adapt cognitive models for large-scale assessment and methods to describe them diagrammatically, as well as systematic records of cognitive models of science learning and research in various academic fields (Leighton & Gierl, 2011). In order to encourage the usage of cognitive models and the subsequent application of AIG, future directions should concentrate on the development of these models, particularly in terms of validity arguments.

## Conclusion

Testing in schools is changing at a never-before-seen pace. AIG is a next-generation assessment process capable of producing numerous high-quality items in a fast and cost-effective manner. Our findings suggest AIG-items are indistinguishable from items manually written by experts, both in terms of structure and psychometric properties. AIG can also allow non-clinical and non-professional item writers to develop items that are comparable to those written by experts. Our findings provide evidence of validity in a number of areas, including construct and internal validity. By using AIG, educational institutions can also reap substantial cost savings without compromising quality. The broader implementation of AIG at medical schools can potentially improve the work of teachers and content experts as well as promote better assessment.

## Figures and Tables

**Fig. 4:**
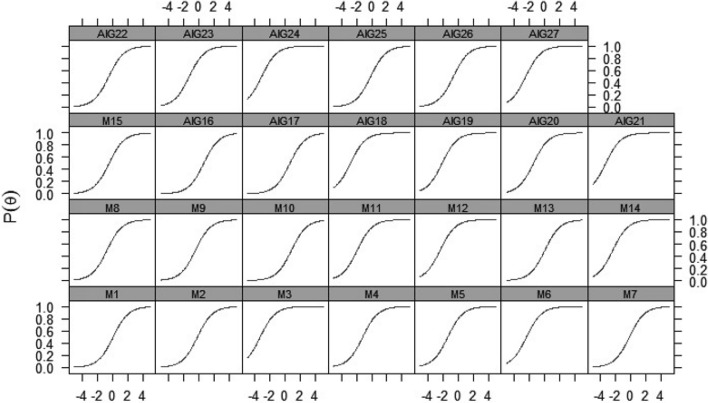
ICCs for the twenty-seven items composing the general and vascular surgery strand

**Fig. 5:**
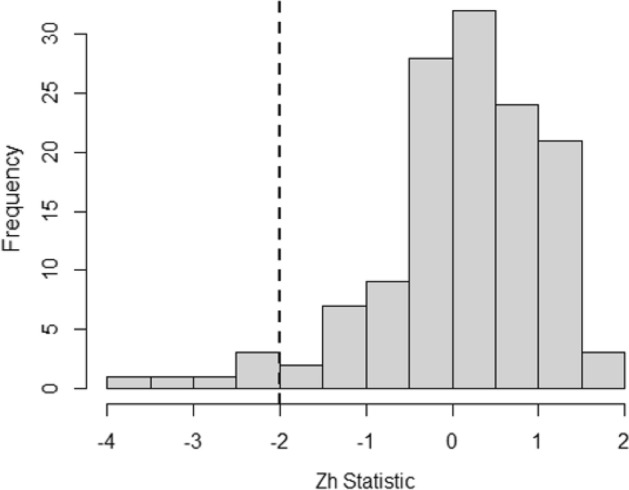
Person fit histogram. *Note*. Large negative values (e.g., Zh <  − 2) indicate person misfit. Large positive values (e.g., Zh > 2) claim that the likelihood of the pattern of the responses is higher than the predictive likelihood of the RM, disturbing latent estimation

**Fig. 6:**
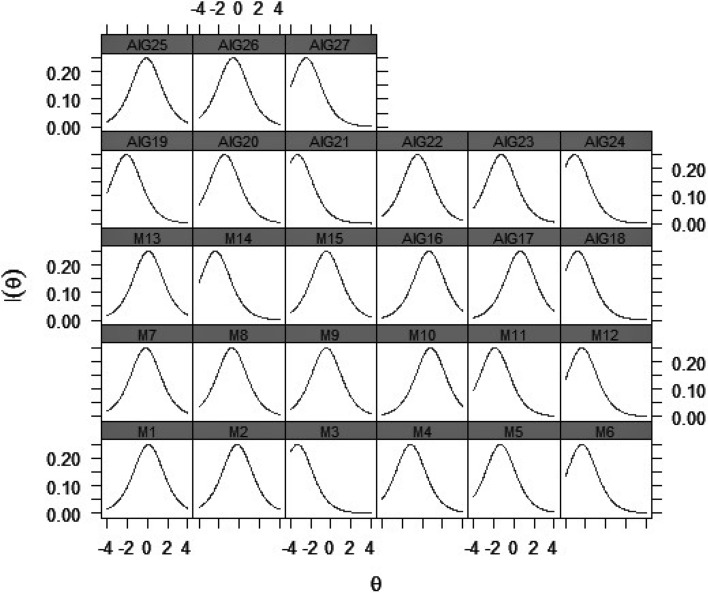
Item information curves plot

## Data Availability

The data that support the findings of this study are available from the corresponding author upon request.

## Appendix A: Learnability analysis

See Table

**Table 1:** ANOVA results using time spent developing the cognitive model (top quadrant) and quantity of items generated (lower quadrant) as the criterion

Source	Sum of Squares	*df*	Mean Square	*F*	*p*	Partial ηp2
(Intercept)	54,159	1	54,159	171	< . 001	0.961
ItemXP	1637	2	818	2.59	0.144	0.425
ClinicXP	583	1	583	1.85	0.216	0.209
ItemXP * ClinicXP	0.626	1	0.626	0.002	0.966	< . 001
Error	2211	7	315			
Intercept	1,442,226	1	1,442,226	56.1	< . 001	.889
ItemXP	634,873	2	317,436	12.3	.005	.779
ClinicXP	354,905	1	354,905	13.8	.008	.663
ItemXP * ClinicXP	419,216	1	419,216	16.3	.005	.700
Error	180,058	7	25,722			

^1^.

## Appendix B: IRT assumptions—strategies and results

*Unidimensionality.* Unidimensionality was evaluated based on two approaches: first, since item response data are rarely strictly unidimensional, it was necessary to consider the existence of additional “minor” abilities along with the main construct of interest (i.e., *knowledge in general and vascular surgery*) (Bonifay et al., 2015). This corresponds to the concept of *essential unidimensionality*, which assumes that a scale is measuring a predominant construct while also tapping into additional factors that are less relevant (Raykov & Pohl, 2013). This hypothesis was assessed using the DETECT index in our study (Stout et al., 1996). The DETECT index is the result of the DETECT, ASSI and RATIO indices, which were calculated for the options unweighted (covariance of item pairs was equally weighted) and weighted (covariance of item pairs was weighted by the sample size of item pairs)(Zhang, 2007). The indices obtained for the weighted and unweighted options were as follows: DETECT = −0.489; ASSI = −0.365; RATIO = −0.498. These values suggest no departure from the assumption of essential unidimensionality (Robitzsch & Robitzsch, 2021).

Second, since we’re analyzing items developed by two different processes, we used two partition criteria of the sample to evaluate if the item parameters were invariant across subgroups splits based on *score* and *gender*. This was evaluated following a consecutive unidimensional approach, more specifically, using the Andersen’s LRT test. For unidimensionality to be met, item parameters based on subgroup fits should be approximately the same. The Andersen LRT test gave a non-significant result for both partition criteria, *score*: χ^2^ = 39.1; df = 26; *p* = 0.047/*gender*: χ^2^ = 24.6; df = 26; *p* = 0.539. No violations of essential unidimensionality were found, providing evidence to support the expect construct in our work. However, one item (AIG 28) was excluded from further analysis due to inappropriate response patterns between person subgroups. The remaining items revealed good fit, and as such provide evidence of internal validity.

*Local independence.* Local independence at the global level was tested through the T11-test. Results suggested that strict local independence did not hold at a global level (*p* < 0.01), meaning that item responses may be correlated or dependent after controlling for one factor. However, these results should not call into question the legitimacy of our analysis. One possible reason for this result may be that the items in the study belong to the same strand in an exam with multiple strands. Furthermore, in most cases, local independence proves to be a difficult assumption to ensure and, for this reason, is often omitted in practice (Mair, 2018). Thus, this assumption is usually subsumed under the unidimensionality assumption(Edelen & Reeve, 2007).

*Monotonicity.* Monotonicity was examined using plots of item characteristic curves (ICCs)—plots of the probability for a test item to be answered correctly, considering the student’s ability and the latent trait being measured (Cf. Figure 4). The probability of responding to an item correctly in this strand was a monotonically increasing function of θ for most items in our exam, corroborating this assumption.

## Appendix C: Model-data fit

We evaluated if the item parameters were invariant across subgroup splits based on score. The Andersen LRT test was non-statistically significant for the score partition criterion (χ^2^ = 39.1; df = 26; p = 0.047), ensuring model fit. Infit and outfit mean square values were close to 1, ensuring item fit. These results represent evidence of internal validity for AIG, as none of its items presented poor fit. The Zh statistic was larger than −2 for most of the respondents, ensuring person fit (Cf. Figure 5).

## Appendix D: RM parameter estimates

See Table

**Table 2:** Rasch Model item parameter estimates and fit statistics for the twenty-seven exam questions

Item	b	SE	χ2	Df	p-value	Outfit MSQ	Infit MSQ
M1	1.29	0.18	129	131	0.52	0.98	0.98
M2	1.01	0.18	152	131	0.10	1.16	1.14
M3	− 2.11	0.44	258	131	0.00	1.96	0.94
M4	0.02	0.21	125	131	0.61	0.95	0.94
M5	− 0.16	0.21	109	131	0.91	0.83	0.89
M6	− 1.17	0.30	114	131	0.85	0.87	0.94
M7	1.01	0.18	126	131	0.58	0.96	0.96
M8	0.48	0.19	161	131	0.04	1.22	1.16
M9	0.75	0.18	130	131	0.50	0.99	1.01
M10	2.02	0.19	116	131	0.82	0.88	0.90
M11	− 0.71	0.25	112	131	0.87	0.85	0.92
M12	− 1.17	0.30	110	131	0.90	0.84	0.92
M13	1.26	0.18	139	131	0.29	1.06	1.05
M14	− 1.17	0.30	113	131	0.86	0.86	0.88
M15	0.75	0.18	143	131	0.22	1.09	1.06
AIG16	1.85	0.19	123	131	0.66	0.94	0.96
AIG17	1.81	0.19	134	131	0.40	1.02	1.01
AIG18	− 1.61	0.36	133	131	0.42	1.01	0.96
AIG19	− 0.92	0.27	91.6	131	1.00	0.69	0.86
AIG20	− 0.26	0.22	150	131	0.12	1.14	1.08
AIG21	− 2.11	0.44	130	131	0.51	0.99	0.96
AIG22	0.72	0.18	132	131	0.44	1.01	0.97
AIG23	− 0.07	0.21	147	131	0.16	1.11	1.03
AIG24	− 1.92	0.41	86.4	131	1.00	0.65	0.90
AIG25	1.07	0.18	112	131	0.88	0.85	0.87
AIG26	0.58	0.19	129	131	0.53	0.98	0.99
AIG27	− 1.27	0.31	109	131	0.91	0.83	0.93

*b*: item difficulty; *SE*: standard error; χ2: Chi-Square item fit statistic; *Df*: degrees of freedom; MSQ: Mean square

2.

## Appendix E: Item information curves

See Fig. 6.

## Footnotes

1 ItemXP of the participants was classified into three different levels (0: “inexperienced”; 1: “Novice”; 2: “experienced”). Members of the staff were classified as inexperienced (n = 3). Clinicians and students were considered as novices (n = 6) since they had knowledge of the MCQs used in tests. Non-clinical teachers and clinical teachers were considered as experienced (n = 3). ClinicXP was treated as a factor with two levels (0: “non-clinic”; 1:”clinic”). Clinicians, clinical-teachers and students from the fifth or sixth year of the medical course at the EMUM were considered as “clinic” (n = 7). Non-clinical teachers, members of the staff and students from the third year were considered as “non-clinic” (n = 5).
